# The effects of alphabetic literacy, linguistic-processing demand and tone type on the dichotic listening of lexical tones

**DOI:** 10.3389/fpsyg.2022.877684

**Published:** 2022-07-26

**Authors:** Jing Shao, Caicai Zhang, Gaoyuan Zhang, Yubin Zhang, Chotiga Pattamadilok

**Affiliations:** ^1^Department of English Language and Literature, Hong Kong Baptist University, Kowloon Tong, Hong Kong SAR, China; ^2^Shenzhen Institutes of Advanced Technology, Chinese Academy of Sciences, Shenzhen, China; ^3^Research Centre for Language, Cognition, and Neuroscience, Department of Chinese and Bilingual Studies, The Hong Kong Polytechnic University, Hung Hom, Hong Kong SAR, China; ^4^Department of Chinese Language and Literature, Peking University, Beijing, China; ^5^Department of Linguistics, University of Southern California, Los Angeles, CA, United States; ^6^Aix Marseille Univ, CNRS, LPL, Laboratoire Parole et Langage, Aix-en-Provence, France

**Keywords:** dichotic listening, alphabetic literacy, linguistic-processing demand, ear preference, lexical tone perception, Cantonese

## Abstract

Brain lateralization of lexical tone processing remains a matter of debate. In this study we used a dichotic listening paradigm to examine the influences of the knowledge of *Jyutping* (a romanization writing system which provides explicit Cantonese tone markers), linguistic-processing demand and tone type on the ear preference pattern of native tone processing in Hong Kong Cantonese speakers. While participants with little knowledge of *Jyutping* showed a previously reported left-ear advantage (LEA), those with a good level of *Jyutping* expertise exhibited either a right-ear advantage or bilateral processing during lexical tone identification and contour tone discrimination, respectively. As for the effect of linguistic-processing demand, while an LEA was found in acoustic/phonetic perception situations, this advantage disappeared and was replaced by a bilateral pattern in conditions that involved a greater extent of linguistic processing, suggesting an increased involvement of the left hemisphere. Regarding the effect of tone type, both groups showed an LEA in level tone discrimination, but only the *Jyutping* group demonstrated a bilateral pattern in contour tone discrimination. Overall, knowledge of written codes of tones, greater degree of linguistic processing and contour tone processing seem to influence the brain lateralization of lexical tone processing in native listeners of Cantonese by increasing the recruitment of the left-hemisphere language network.

## Introduction

### Ear preference in lexical tone perception revealed by dichotic listening studies

Over the past few decades, numerous studies have shown that the human brain is functionally specialized. A left-hemisphere (LH) specialization has been found for verbal material processing (Kimura, 1967; Hugdahl et al., 1999) and a right-hemisphere (RH) specialization for nonverbal material processing (Boucher and Bryden, 1997; Hugdahl et al., 1999). Dichotic listening, in which different auditory stimuli are presented simultaneously to left and right ears, is a neuropsychological technique for studying perceptual laterality. Findings have shown a right-ear advantage (REA) for the processing of spoken syllables and digits, indicating an LH dominance (Kimura, 1961, 1967; Studdert-Kennedy and Shankweiler, 1970; Hugdahl et al., 1999), and a left-ear advantage (LEA) for music and pitch processing (Wioland et al., 1999; Brancucci et al., 2005; Hoch and Tillmann, 2010), indicating an RH dominance.

Since the 19th century, the REA has been thought to be dominant in perception of segmental speech input such as consonants and vowels (Cutting, 1974; Dwyer et al., 1982; Bryden and Murray, 1985). However, the ear preference and its underlying brain lateralization of suprasegmental elements like lexical tones are still an issue of debate. Two hypotheses have been put forward to explain the brain lateralization patterns in lexical tone processing, i.e., the functional hypothesis and the acoustic hypothesis. The functional hypothesis (Van Lancker, 1980; Whalen and Liberman, 1987; Gandour Wong and Hutchins, 1998; Gandour et al., 2000; Liberman and Whalen, 2000) assumes that brain lateralization is dependent on the functional role of the auditory signal. This view predicts that speech stimuli are primarily processed in the LH, typically considered as the dominant hemisphere for language processing in right-handed individuals, whereas non-speech signals are processed primarily in the RH. On the other hand, the acoustic hypothesis (Zatorre and Belin, 2001; Zatorre et al., 2002; Poeppel, 2003) claims that the acoustic structures of auditory inputs determine the brain lateralization: spectral processing, including pitch-related and suprasegmental information, is lateralized to the RH, whereas fast temporal processing, such as segmental information, induces more LH activation. Given the unique nature of lexical tones, the functional and acoustic hypotheses make diverging predictions. The functional hypothesis predicts an LH dominance in native speakers of tonal languages, based on their linguistic functions; however, the acoustic hypothesis predicts an RH dominance for processing lexical tones, based on their acoustic features.

While dichotic listening studies on native tone perception have generated empirical support for both hypotheses (Wang et al., 2001; Luo et al., 2006; Jia et al., 2013), the actual lateralization patterns are more complex than those predicted by the two hypotheses, and appear to vary across languages. In summary, three distinct patterns of brain lateralization of lexical tones have been revealed by dichotic listening studies: (1) an REA in processing lexical tones by native Mandarin Chinese, Thai and Norwegian speakers (Van Lancker and Fromkin, 1973, 1978; Moen, 1993; Wang et al., 2001); (2) bilateral processing by native Mandarin Chinese speakers (Baudoin-Chial, 1986); (3) an LEA in the perception of lexical tones by native Hong Kong Cantonese speakers (Jia et al., 2013), regardless of the tone type (level tones and contour tones), stimulus type (hums, real syllables and pseudosyllables) and task (discrimination task and identification task). Of particular note is the last pattern reported in Hong Kong Cantonese, which diverges from those observed in Thai, Norwegian and Mandarin Chinese speakers, and further deviates from the convergent finding of LH activation in tone processing in native tonal language listeners as revealed by a meta-analysis on neuroimaging studies (Liang and Du, 2018). This discrepancy across studies indicates that additional factors may influence the hemispheric laterality of native tone processing other than the functional and acoustic explanations.

One possible factor is the lack of training in a native alphabetic script of spoken Cantonese in Hong Kong Cantonese speakers, as also argued by Jia et al. (2013). In contrast to Mandarin Chinese and Thai in which tones are marked as written labels with various degrees of precision in the respective alphabet (*Pinyin* or the Thai script), most Hong Kong Cantonese speakers learned logographic Chinese without exposure to a native alphabetic script of spoken Cantonese. Based on the well-established link between alphabetic literacy and phonological awareness, including tone awareness (Morais et al., 1979; Cheung et al., 2001; Shu et al., 2008; Li and Suk-Han Ho, 2011), lack of exposure to written codes of the native tones may account for the LEA observed in Hong Kong Cantonese listeners. Other factors that may explain the discrepancy are the differences in stimulus type (e.g., nonspeech and speech tones) and tone type (e.g., level tones and contour tones). Thus, the first and primary aim of this study is to examine whether knowledge of a native alphabetic script with codes for lexical tones would influence the ear preference pattern of lexical tone processing in Cantonese listeners. The second aim is to further investigate the influences of linguistic-processing demand and tone type on dichotic listening of lexical tones, and the potential impact of the knowledge of tone markers on these two factors.

#### The effect of alphabetic literacy

It has been well established that learning an alphabetic script boosts phonological awareness at the behavioral level (Morais et al., 1979; Cheung et al., 2001; Shu et al., 2008; Zhang Y. et al., 2021). Phonological awareness usually refers to the ability to analyze the spoken language into smaller units such as phonemes (Liberman et al., 1974). Since lexical tone is a suprasegmental unit that distinguishes word meanings in tonal languages, tone awareness can be considered as a component of phonological awareness, which involves the ability to recognize and extract the lexical tone from a speech unit (Shu et al., 2008; Li and Suk-Han Ho, 2011). For example, Cheung et al. (2001) examined the development of phonological awareness in Hong Kong and Guangzhou children who both spoke Cantonese. Guangzhou children usually had early experience with alphabetic Chinese reading (*Pinyin*) in addition to logographic Chinese reading, whereas Hong Kong children read only logographic Chinese. The results revealed that at the pre-reading stage, the Hong Kong and Guangzhou children showed similar performance on phonological awareness. However, after learning to read, the Guangzhou children outperformed their Hong Kong counterparts on several phonological awareness tasks, indicating that learning *Pinyin* boosts phonological awareness. With regard to tone awareness, Shu et al. (2008) reported that phonological coding (*Pinyin*) instruction, which provides explicit markers for individual lexical tones, significantly improved this ability in Mandarin-speaking children: Tone awareness arose from chance level in preschoolers to over 74% accuracy in first graders after receiving *Pinyin* instruction. Although tones are marked with non-alphabetic symbols (e.g., diacritics in *Pinyin*), the development of tone awareness is presumably governed by the same principle that supports the development of phonemic awareness at the segmental level when one learns to read in an alphabetic system (Morais et al., 1979; Read et al., 1986; Morais, 2021).

At the neural level, learning an alphabetic script has been reported to induce functional reorganization of the LH language network (Dehaene et al., 2010; Brennan et al., 2013). Brennan et al. (2013) conducted a study that examined the influence of learning to read on brain activities associated with spoken language processing in English and Chinese speakers. The authors reported that, compared to Chinese speakers who had much less experience with an alphabetic writing system, English speakers showed developmental increases of brain activity in the LH phonological network, including the superior temporal gyrus, inferior parietal lobule and inferior frontal gyrus. These findings led the authors to conclude that learning to read in an alphabetic writing system reorganizes the phonological awareness network, and that such reorganization might lead to better phonological awareness skills.

In light of the aforementioned impacts of alphabetic literacy on boosting tone awareness and reorganizing the LH phonological network, we hypothesized that Cantonese listeners with knowledge of written codes that allow clear identification of the individual lexical tones present in the spoken language would demonstrate greater REA in native tone processing compared to those without such knowledge. Studying tone processing in Cantonese allowed us to further investigate the RH dominance of tone processing in Hong Kong Cantonese speakers, which has so far been reported only by Jia et al. (2013). Additionally, it also allowed us to avoid possible confounds related to age, education level or the maturation of the spoken language system, that may occur when the same question is addressed, for instance, by comparing performance in young native Mandarin Chinese speakers with different levels of *Pinyin* skills. Indeed, in contrast to the widespread *Pinyin* instruction that typically starts in the primary school in the mainland, the majority of Hong Kong Cantonese speakers learned logographic Chinese without being exposed to a native alphabetic script of spoken Cantonese. Although many Cantonese speakers in Hong Kong also learned English and its alphabetic script from childhood, tones are not coded in the English script, and it is highly unlikely that proficiency in the English script would boost tone awareness in Cantonese speakers (Bialystok et al., 2005; Dodd et al., 2008; Deng et al., 2019). Furthermore, as an intonation language, English uses large (coarse-grained) pitch modulations to index intonation differences (statement/question) and lexical stress, whereas smaller (more refined) pitch modulations are used to differentiate lexical tones in Cantonese (Liu et al., 2010), which would make any transfer of English phonological knowledge to tone awareness in Cantonese difficult. Despite the lack of an official Cantonese alphabetic script, several non-standard Cantonese romanization systems are concurrently in use in Hong Kong, primarily as online Chinese input methods. Among these systems, *Jyutping* is one of the few systems that provide a precise coding of lexical tones. *Jyutping* is a romanization system of spoken Cantonese devised by the Linguistic Society of Hong Kong in 1993. Tones are transcribed in *Jyutping* as numbers 1–6 (e.g., ‘變化’ bin3 faa3 ‘change’, with ‘3’ indicating the third tone in Cantonese), which correspond to the six tones in Cantonese: T1 /55/ high level tone, T2 /25/ high rising tone, T3 /33/ mid-level tone, T4 /21/ low falling/extra low level tone, T5 /23/ low rising tone, and T6 /22/ low level tone (Bauer and Benedict, 1997). Thus, *Jyutping* is particularly suitable for examining the ear preference pattern of lexical tone processing in Cantonese listeners.

#### The effect of linguistic-processing demand

The second issue addressed in this study is the role of linguistic-processing demand on the modulation of ear preference patterns in dichotic listening of lexical tones. Acoustically, lexical tones result from a modulation of pitch contour. At the same time, it also serves as a distinctive feature for distinguishing word meanings in tonal languages, comparable to the role of phonemes. As mentioned above, these unique characteristics of lexical tone might have led to the mixed findings regarding its ear preference and brain lateralization: At the acoustic level, it may be mainly processed based on its acoustic features and leads to an ear preference pattern similar to that of prosody. At the phonological level, it may elicit an ear preference pattern resembling that of phonemes. Indeed, as revealed by Liang and Du (2018) using a meta-analysis approach, lexical tones, like prosody, showed more extensive activations in the right than the left auditory cortex in both tonal and non-tonal language speakers, whereas the LH was recruited during lexical tone processing exclusively by native tonal language speakers, consistent with the activation pattern of phonemes. In other words, it is likely that lexical tones induce RH activation in both tonal and non-tonal language speakers due to their low-level acoustic feature, whereas tonal language speakers additionally engage the LH because lexical tones are further processed as a phonological unit.

In addition to the role of tonal vs. non-tonal language experience, previous studies have revealed that even within native tonal language speakers, different amounts of linguistic information contained in the stimuli or different degrees of linguistic processing elicited different ear preference patterns (Shuai and Gong, 2014; Mei et al., 2020). According to an EEG study using the dichotic listening paradigm, auditory processing of pitch variations elicited greater activation of the RH as a bottom-up effect; linguistic processing of lexical tones, on the other hand, evoked greater activation of the LH as a top-down effect (Shuai, 2009; Shuai and Gong, 2014). Mei et al. (2020) reported that when the stimuli contained slow frequency modulation of tones only, such as in hums, an LEA was observed in native Mandarin listeners. However, when the stimuli contained linguistic cues such as phoneme and lexical information (e.g., in the condition where lexical tones were carried by a single vowel), the LEA was likely to disappear. Furthermore, bilateral processing was found when more phonological and lexical-semantic attributes were included, as in the consonant-vowel (CV), pseudo-word, and word conditions. These findings suggest that linguistic complexity of the stimuli and linguistic processing demand contribute to the brain specialization of lexical tones.

Based on the above existing observations, the second aim of this study is to further test the hypothesis that different degrees of linguistic processing result in different ear preference patterns, by employing three types of stimuli to vary the degree of linguistic processing—non-speech stimuli, speech stimuli with low syllable variation and speech stimuli with high syllable variation (see descriptions in *The Current Study* below).

#### The effect of tone type

The last aim of the current study is to revisit the influence of tone type on ear preference. The Cantonese tonal system can be classified as comprising three static level tones that primarily differ in pitch height (T1 – high level tone, T3 – mid level tone, and T6 – low level tone) and three dynamic contour tones that primarily differ in the direction of pitch change (T2 – high rising tone, T4 – extra low level/low falling tone, and T5 – low rising tone; Bauer and Benedict, 1997). Whereas pitch height is the primary cue for distinguishing the three level tones with discernible pitch differences from the pitch onset, both pitch height and direction are involved in contour tone distinction and pitch cues in the later portion of the pitch curve might be more critical for contour tone perception (Khouw and Ciocca, 2007). It has been found that native Cantonese listeners placed more weight on or were more sensitive to pitch height than pitch contour cues (Gandour, 1983; Khouw and Ciocca, 2007; Jia et al., 2013; Zhang C. et al., 2021). Accordingly, a study showed that the accuracy in the perception of three level tones was higher compared to the more complex contour tones (Jia et al., 2013). Early processing of these two types of tones were also found to be different, in that they elicited different ERP components, namely, a prominent MMN in level tones but a P3a in contour tones in Cantonese listeners (Tsang et al., 2011). Altogether, these observations point out processing differences between level and contour tones.

According to the temporal integration hypothesis (Poeppel, 2003; Boemio et al., 2005; Sanders and Poeppel, 2007; Teng et al., 2016; Flinker et al., 2019), the left and right auditory cortices have differential sensitivity towards acoustic information over varied time-scales: whereas the left auditory cortex (AC) preferentially processes information from short temporal integration windows (25–50 ms), the right AC preferentially processes information from long temporal integration windows (200–300 ms). In light of this hypothesis, it is likely that the fast-changing and dynamic contour tones would require the extraction of pitch information over short temporal windows (Krishnan and Gandour, 2009), increasing the LH participation, in contrast to the slowly-changing and static level tones. In addition, as mentioned above, the dynamic contour tones that are perceptually more challenging might require deeper processing, which may also increase the LH participation.

However, mixed findings have been reported regarding the ear preference pattern of contour vs. level tone processing (Ho, 2010; Jia et al., 2013). Although Ho (2010) found that pitch height and contour changes induced different hemispheric advantages, both level and contour tones were reported to elicit a greater RH advantage in Jia et al. (2013). We aimed to revisit the influence of tone type on ear preference and further examine whether the effect of tone type interacted with that of *Jyutping* expertise on ear preference in the current study.

### The current study

In the present study, we examined the impacts of three factors – *Jyutping* expertise (*Jyutping* vs. non-*Jyutping* group), linguistic-processing demand (nonspeech vs. low syllable variation vs. high syllable variation) and tone type (level vs. contour tones), as well as their interactions on ear preference of lexical tone processing in native Cantonese speakers using the dichotic listening paradigm. As with the previous study on Cantonese (Jia et al., 2013), we employed an identification task and a discrimination task to examine the dichotic listening of lexical tones. In the current study, the effect of *Jyutping* expertise was investigated *via* a comparison of two matched groups of native Cantonese speakers without *Jyutping* expertise (henceforth, non-*Jyutping* participants) and with *Jyutping* expertise (henceforth, *Jyutping* participants), in order to elucidate the influence of knowledge of Cantonese tonal codes on the ear preference of lexical tone processing. The impact of linguistic-processing demand was examined by three stimulus types—nonspeech tones, speech materials with low syllable variation and speech materials with high syllable variation. The nonspeech tone condition, which only contained pitch information extracted from the speech materials, was included to induce primarily acoustic processing of lexical tones (Van Lancker and Fromkin, 1973; Shuai and Gong, 2014; Mei et al., 2020). The low and high variation conditions both used meaningful Cantonese words and would engage more linguistic (e.g., phonological and lexical) processing relative to the nonspeech condition. The critical difference between these two conditions concerned the carrying syllables in a dichotic pair, which remained constant in the low variation condition (e.g., /ji55/ ‘doctor’ – /ji22/ ‘second’), but varied in the high variation condition (e.g., /ji55/ ‘doctor’ – /fɐn22/ ‘part’). Note that the two stimuli in a dichotic pair involved a meaning change in both low and high variation conditions, and thus the two conditions may be deemed to be largely matched in this regard, constraining the primary difference between them to the changing of carrying syllables. Previous studies have indicated that different degrees of syllable variability may tap into different levels of linguistic processing (Lee et al., 2008; Shu et al., 2008; Shao et al., 2019). In the low variation condition, tone perception could be carried out primarily by comparing the acoustic forms of the stimuli without having to segregate the tone from the segmental units (Burton et al., 2000), thus requiring relatively less phonological processing. In contrast, in the high variation condition, initial separation between segmental and suprasegmental units seems necessary before conducting the comparison of tone categories (Burton et al., 2000), demanding greater efforts in phonological processing. Moreover, this meta-phonological ability has been reported to develop with language experience and reading ability (Shu et al., 2008; Zhang Y. et al., 2021). Therefore, we employed these three types of stimuli to further test the hypothesis that different degrees of linguistic processing, especially phonological segmentation demand, influence the patterns of ear preference. Lastly, we investigated the effects of tone type by comparing the processing of three level tones (T1, T3 and T6) versus three contour tones (T2, T4 and T5) in Cantonese.

With regard to the effect of *Jyutping* expertise, based on previous observations that alphabetic literacy boosts phonological awareness (including tone awareness) and induces the activation of the LH phonological network (Cheung et al., 2001; Shu et al., 2008; Dehaene et al., 2010; Brennan et al., 2013; Zhang Y. et al., 2021), we predicted that participants with *Jyutping* knowledge may show greater REA (LH dominance) in the dichotic listening of lexical tones compared to their non-*Jyutping* peers. Regarding the effects of linguistic-processing demand, we predicted that situations that place a higher demand on linguistic processing, especially phonological segmentation (e.g., the high variation condition), would lead to greater engagement of the LH, yielding bilateral processing or even REA. On the contrary, an LEA was expected in the conditions that required minimal linguistic processing (e.g., the nonspeech condition). In terms of the effect of tone type, level tones are expected to elicit an LEA, in contrast to contour tones that may exhibit more bilateral processing or even REA. Finally, we also explored whether there would be an interaction between *Jyutping* expertise and the effects of linguistic-processing demand and tone type. It is possible that participants with knowledge of *Jyutping* are likely to show an REA especially in the processing conditions that require more LH engagement.

## Materials and methods

### Participants

Eighteen non-*Jyutping* participants (8 M, 10F) and 16 *Jyutping* participants (9 M, 7F) were recruited based on their self-report of *Jyutping* knowledge which was confirmed by a *Jyutping* transcription test (see below). All the participants were native speakers of Hong Kong Cantonese. The participants were pre-screened based on the criteria of being right-handed as assessed by the Edinburgh Handedness Inventory (Oldfield, 1971), having no hearing impairment, and having no formal musical training. Participants with linguistics background were deliberately excluded. The two groups were largely matched in age (*Jyutping*: mean = 22.2, age range = 20–24; non-*Jyutping*: mean = 22.3, age range = 18–28) and education level. The *Jyutping* proficiency (or lack of it) of the two groups of participants was confirmed using a timed *Jyutping* transcription test, which contained 20 disyllabic words that covered the full Cantonese phonetic inventory. The 20 words were presented in Chinese characters on a piece of paper, and the participants were instructed to write down the *Jyutping* transcriptions of these words (including tones) as fast as possible. The participants without *Jyutping* knowledge were instructed to skip the trials or guess the transcriptions. Only the participants who scored above 50% accuracy in tone transcription were included in the *Jyutping* group. Accuracy of tone transcription in the *Jyutping* group was significantly higher than the non-*Jyutping* group (*t*(33) = −9.539, *p* < 0.001; *Jyutping*: *M* = 72.94%, *SD* = 13.8%; non-*Jyutping*: *M* = 29.13%, *SD* = 15.1%).

The experimental procedure was approved by the Human Subjects Ethics Sub-committee of The Hong Kong Polytechnic University (Application number: HSEARS20190502004). Informed written consent was obtained from the participants in compliance with the experiment protocols.

### Stimuli

There were three stimulus conditions: nonspeech tone, low variation and high variation conditions. The stimuli used in the low variation condition were six words contrasting six Cantonese tones on the syllable /ji/. The stimuli used in the high variation condition were 18 words contrasting six Cantonese tones on the syllables /fɐn/, /jɐu/, and /wɐi/ (see Table 1). In addition to these critical stimuli that were used in both identification and discrimination task, six tones carried by the base syllable /ŋa/ were employed as mask items in the discrimination task (see further details in Procedure). These base syllables were selected because they can yield meaningful morphemes in combination with every tone, which enables us to have a full tonal coverage while controlling for the base syllable variability (5 syllables × 6 tones). All the syllables are free or bound morphemes. While syllables carrying T1, T3, and T6 were grouped into the level tone condition, syllables carrying T2, T4, and T5 were grouped into the contour tone condition.

**Table 1:** The five sets of syllables used in the experiment.

	T1 high level /55/	T2 high rising /25/	T3 mid-level /33/	T4 low falling /21/	T5 low rising /23/	T6 low level /22/
/ji/	醫 “doctor”	椅 “chair”	意 “meaning”	兒 “son”	耳 “ear”	二 “two”
/fɐn/	婚 “marriage”	粉 “pink”	訓 “train”	焚 “burn”	奮 “strive”	份 “part”
/wɐi/	威 “power”	委 “council”	餵 “feed”	圍 “surround”	偉 “grand”	胃 “stomach”
/jɐu/	休 “rest”	黝 “dark”	幼 “young”	油 “oil”	友 “friend”	右 “right”
/ŋa/	鴉 “crow”	啞 “mute”	亞 “Asia”	牙 “teeth”	雅 “proper”	訝 “astonished”

One female native Cantonese speaker was recorded reading aloud these words in a carrier sentence, 呢個字係 /li55 ko33 tsi22 hɐi22/ (‘This word is’) in a clear and deliberate manner. Each sentence was recorded six times. Then, the most clearly produced token was selected and the word was segmented out of the carrier sentence. All selected words were normalized such that they had the same acoustic intensity (60 dB) and duration (620 ms, which corresponded to the mean duration of all selected words; Praat: Boersma and Weenink, 2014). The first author checked the naturalness of the stimuli after normalization.

The nonspeech tone stimuli were nonspeech analogues of the stimuli that were used in the low variation condition. A 620-ms pure tone sound was first generated using Praat, and then a total of 12 F0 contours of the syllables /ji/ and /ŋa/ were extracted and superimposed on the pure tone sound, generating 12 pure tone stimuli. The mean acoustic intensity of the pure tone stimuli was set to 75 dB, which was 15 dB louder than the speech stimuli. This adjustment allowed us to match the subjective intensity of the two types of stimuli (Zhang et al., 2017; Shao and Zhang, 2020).

### Procedure

Both identification and discrimination tasks were employed in a total of six conditions as defined by the stimulus type and tone type (3 × 2). The design of the identification task was adopted from Jia et al. (2013). In each trial, there was a dichotic pair presented to the two ears simultaneously. Level tones and contour tones were presented in separate blocks. For both tone types, there were three same-tone pairs and six different-tone pairs. For the level tones, the same-tone pairs included T1-T1, T3-T3, and T6-T6, while the different-tone pairs included T1-T3, T1-T6, T3-T6, T3-T1, T6-T1, and T6-T3. For the contour tones, the same-tone pairs included T2-T2, T4-T4, and T5-T5, while the different-tone pairs included T2-T5, T5-T2, T4-T5, T5-T4, T4-T2, and T2-T4. In the low variation condition, the two items within each trial had the same base syllable (e.g., /ji55/−/ji33/). In the high variation condition, the base syllables differed (e.g., /fɐn55/−/jɐu33/). Note that the three syllables formed three pairs of syllables (fɐn/−/jɐu/, /fɐn/−/wɐi/ and /jɐu/−/wɐi/) with equal probabilities to occur. In the nonspeech tone condition, all the stimuli were pure tones with the same F0 trajectories as the stimuli used in the low variation condition. For all stimulus types, the task was to identify the most clearly heard tone (presented to either the left or right ear) by pressing the button on the keyboard as soon as possible, i.e., 1–6 (corresponding to T1 to T6) within 5 s. The identification task contained six blocks in total, which corresponded to the combination of the two tone types and the three stimulus types. Within each block, the three same pairs were repeated six times and the six different pairs were also repeated six times, generating a total of 54 trials. The presentation of stimulus pairs was randomized. In this task, we did not balance the number of same and different pairs, because identical tones were presented to both ears in the same pairs, which did not probe dichotic listening and was not our primary interest. Each block lasted about 3–3.5 min and the whole identification task took about 20 min. Participants were asked to take a five-minute break every three blocks.

The design of the discrimination task followed that of Brancucci et al. (2008) and Jia et al. (2013). Within each trial, two dichotic pairs were consecutively presented. The participants were instructed to direct their attention to a designated ear (i.e., the testing ear). The first pair composed of a target and a mask. The second pair composed of a probe and a mask. The target and probe were always presented in the testing ear; the mask was always presented in the ear to be ignored. The task was to judge whether the target and the probe were, or not, pronounced with the same tone as soon as possible within 3 s, by pressing the button on the keyboard (“left arrow” if same, and “right arrow” if different).

In the nonspeech tone condition, the masks were pure tones with the same F0 trajectories as the syllable /ŋa/, and the targets and probes were pure tones carrying the same F0 trajectories as the syllable /ji/. In the low and high variation conditions, the masks were words with the syllable /ŋa/. The carrying syllables for the targets and probes for the low variation condition was /ji/. For the high variation condition, they were /fɐn/, /jɐu/, and /wɐi/. These syllables were grouped into three pairs (/fɐn/−/jɐu/, /fɐn/−/wɐi/ and /jɐu/−/wɐi/), which had equal chance to occur. The discrimination task contained 12 blocks in total, which corresponded to the combination of the two tone types, the three stimulus types and the two testing ears. Within each block, the sequences of stimulus presentation were randomized. The same pairs were repeated six times (3 × 6), and different pairs were repeated three times (6 × 3), creating equal numbers of same and different pairs in each block. Each block lasted about 2–2.5 min and the whole discrimination task took about 25 min. Participants were asked to take a five-minute break every three blocks.

The tasks were programmed with E-prime 1.0 (Psychology Software Tools, Pittsburgh, PA). All the tasks were conducted in a soundproof booth in the Speech and Language Sciences Lab at the Hong Kong Polytechnic University. The stimuli were presented *via* headphones to the participants at a comfortable listening level. The volume level was kept constant across all the tasks within each participant. No participants reported any difficulty hearing the stimuli. The discrimination task required the participants to direct their attention to a specific ear in each block, whereas the identification task did not. To avoid the effect of direction of attention transferring from the discrimination task to the identification task, all participants completed the identification task before the discrimination task. Before each task, a practice session was provided to familiarize the participants with the procedure and to ensure that they fully understood the instruction. No feedback was given during the practice sessions. The presentation order of blocks within each task was counterbalanced across participants.

### Data analysis

We measured both accuracy and reaction time (RT) to examine the hemispheric lateralization pattern, following previous studies (Jia et al., 2013; Reilly et al., 2015). Linear mixed-effects (LME) analyses were performed on the R platform using the *lme4* (Bates et al., 2014), *lmerTest* (Kuznetsova et al., 2017), and *emmeans* packages (Lenth and Lenth, 2018). The *anova* function of the R package was used to obtain the *p* values of the main effects and the interactions in the models. The *emmeans* package was used to conduct pairwise comparisons with Tukey’s correction.

Identification accuracy was computed as the relative portion of the correct responses in each ear. The maximal model was first fitted using the following variables and their interactions: *group* (*Jyutping* vs. non-*Jyutping*), *stimulus type* (nonspeech tone vs. low syllable variation vs. high syllable variation), *tone type* (level tone vs. contour tone) and *ear* (left ear vs. right ear). The random intercepts of subjects, along with the random slope of the interaction between categories, stimulus type and ear per subject were treated as the random factors.^1^ To reach a simpler model, the random intercepts and slopes were removed one by one using the likelihood ratio test (LRT) in R. The same method was used to remove the least contributing predictors in terms of fixed factors.

Regarding the discrimination accuracy, generalized mixed-effects models were fitted on the responses to each trial (correct response was coded as “1” and incorrect response was coded as “0”). The fixed effects were *group*, *stimulus type, tone type, ear* and *their interactions*. The random intercepts of subjects and stimulus, along with the random slope of the interaction between categories, stimulus type and ear per subject and random slope of group per stimulus were treated as the random factors. The same model comparison procedure as in the identification task was applied.

In the RT analyses, identification RT was measured from the offset of the stimuli to the time that a response was made. RT in the discrimination task was measured from the offset of the second dichotic pair to the time that a response was made. In both tasks, trials with null and incorrect responses were excluded from the analysis and RT data of the remaining trials were log-transformed. Linear mixed-effects models were fitted with the fixed effects including *group*, *stimulus type, tone type, ear* and *their interactions*. The random intercepts of subjects and stimulus, along with the random slope of the interaction between categories, stimulus type and ear per subject and random slope of group per stimulus were treated as the random factors. The same model comparison procedure described was applied. Given that these analyses yielded a number of main effects and interactions, for the sake of simplicity, the results are presented in different sub-sections that correspond to the research questions posed in the Introduction. Unless stated otherwise, only the significant effects are reported. The full results of the models are reported in the supplemental materials.

## Results

### Does jyutping expertise have an influence on ear preference of lexical tone processing?

**Figure 1:**
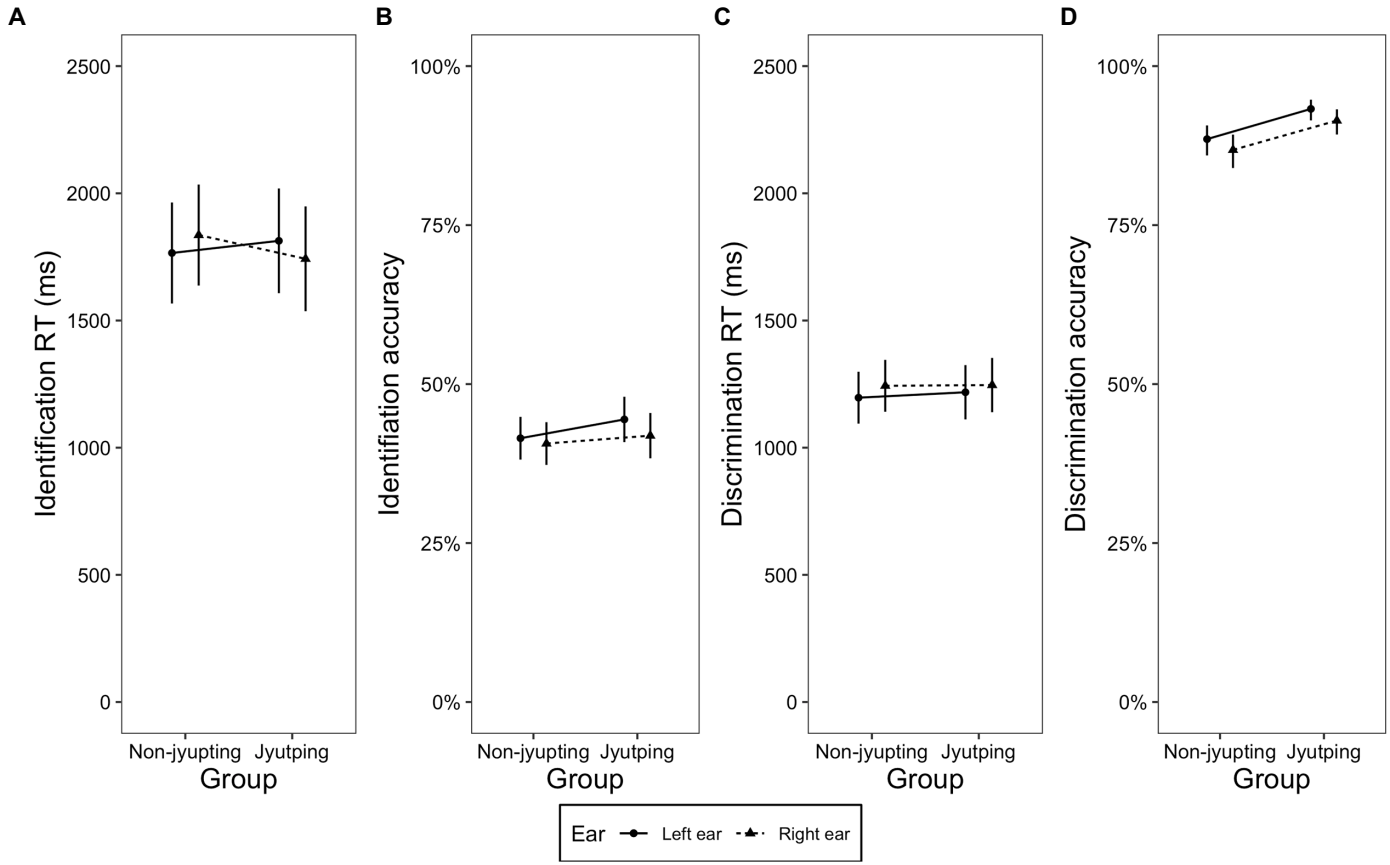
Plots displaying the interaction of ear and group. **(A)** Identification RT, **(B)** identification accuracy, **(C)** discrimination RT, and **(D)** discrimination accuracy of the stimuli presented in the left and right ear in the *Jyutping* and non-*Jyutping* participants. The error bars indicate 95% confidence interval.

This research question was addressed by examining the presence or absence of the interaction between *ear* and *group*.^2^,^3^ Figures 1A–D displays the identification RT, identification accuracy, discrimination RT and discrimination accuracy in terms of *ear* and *group*. Among the analyses on the RTs and accuracy scores of the identification and discrimination tasks, the interaction was significant only in the RT analysis of the identification task [*F*(2, 5,897) = 4.39, *p = 0*.01]. Pairwise comparisons examining the *ear* effect in each group of participants showed that, in the non-*Jyutping* group, RTs obtained on the stimuli presented in the left ear were significantly shorter than RTs obtained on stimuli presented in the right ear (Estimate = −0.0160, Std. Error = 0.007, *t* = −2.198, *p* = 0.028), suggesting an LEA. The *Jyutping* group showed the opposite pattern: The RTs obtained on stimuli presented in the right ear were significantly shorter (Estimate = 0.0203, Std. Error = 0.007, *t* = 2.710, *p* = 0.006), suggesting an REA in the *Jyutping* group (Figure 1A).

### Do linguistic-processing demand and tone type have an influence on ear preference of lexical tone processing?

**Figure 2:**
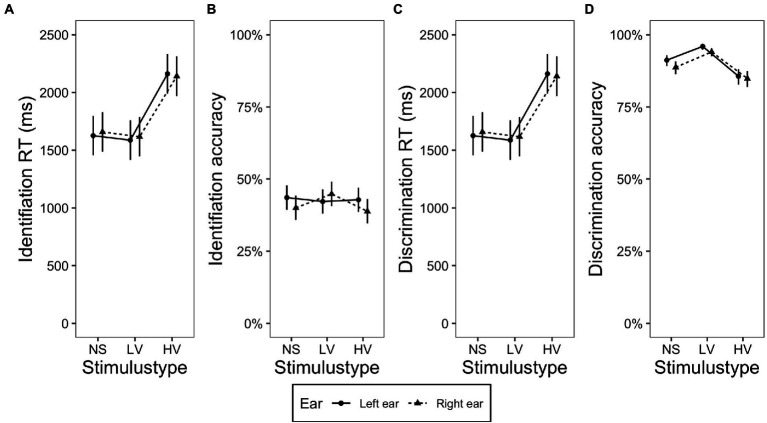
Plots displaying the interaction of stimulus type and ear. **(A)** Identification RT, **(B)** identification accuracy, **(C)** discrimination RT, and **(D)** discrimination accuracy obtained on the stimuli presented in the left and right ear in the nonspeech tone (NS), low variation (LV) and high variation (HV) conditions. The error bars indicate 95% confidence interval.

**Figure 3:**
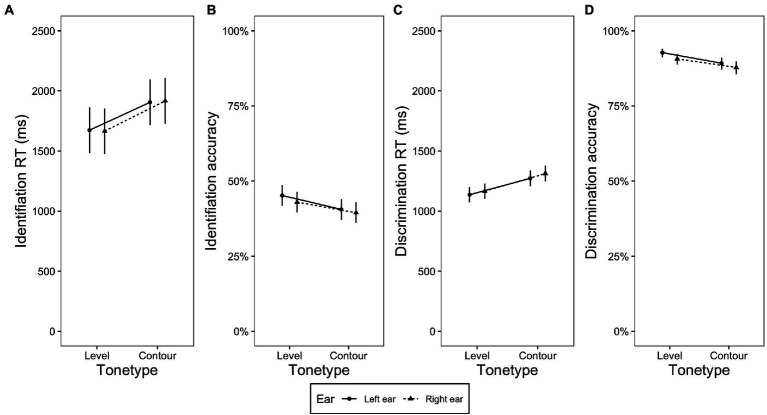
Plots displaying the interaction of tone type and ear. **(A)** Identification RT, **(B)** identification accuracy, **(C)** discrimination RT, and **(D)** discrimination accuracy obtained on the level and contour tones presented in the left and right ear. The error bars indicate 95% confidence interval.

These two research questions were addressed by examining the presence or absence of the interaction between *ear* and linguistic-processing demand (*stimulus type*) or *tone type*. Since there were not many significant effects in relation to these two questions, they are reported together in this section. Figures 2A–D, 3A–D displays the identification RT, identification accuracy, discrimination RT and discrimination accuracy in terms of the interaction of *stimulus type* and *ear*, and the interaction of *tone type* and *ear*, respectively.

Regarding the influence of *stimulus type*, analysis on discrimination accuracy revealed a significant two-way interaction between *stimulus type* and *ear* [*χ*^2^(4) = 92.91, *p* < 0.001; Figure 2D]. Pairwise comparisons showed that accuracy scores on stimuli presented in the left ear were higher than scores on stimuli presented in the right ear in the nonspeech tone condition (Estimate = 0.325, Std. Error = 0.1, *z* = 3.251, *p* < 0.01) and low variation condition (Estimate = 0.428, Std. Error = 0.139, *z* = 3.080, *p* < 0.001), but not in the high variation condition (Estimate = 0.063, Std. Error = 0.07, *z* = 0.724, *p* = 0.47), indicating that a significant LEA was observed in the nonspeech and low variation conditions, whereas the high variation condition showed a bilateral pattern. No two-way interaction between *ear* and *stimulus type* was found in the other analyses (*p*s > 0.05, see Figure 2).

Regarding the influence of *tone type*, no two-way interaction between *ear* and *tone type* was found (*p*s > 0.05, see Figure 3).

### Does the interaction between Jyutping expertise, linguistic-processing demand and tone type influence ear preference on lexical tone processing?

This section examined the existence of complex interaction patterns between *ear*, *group* and linguistic-processing demand (*stimulus type*) and *tone type*.

**Figure 4:**
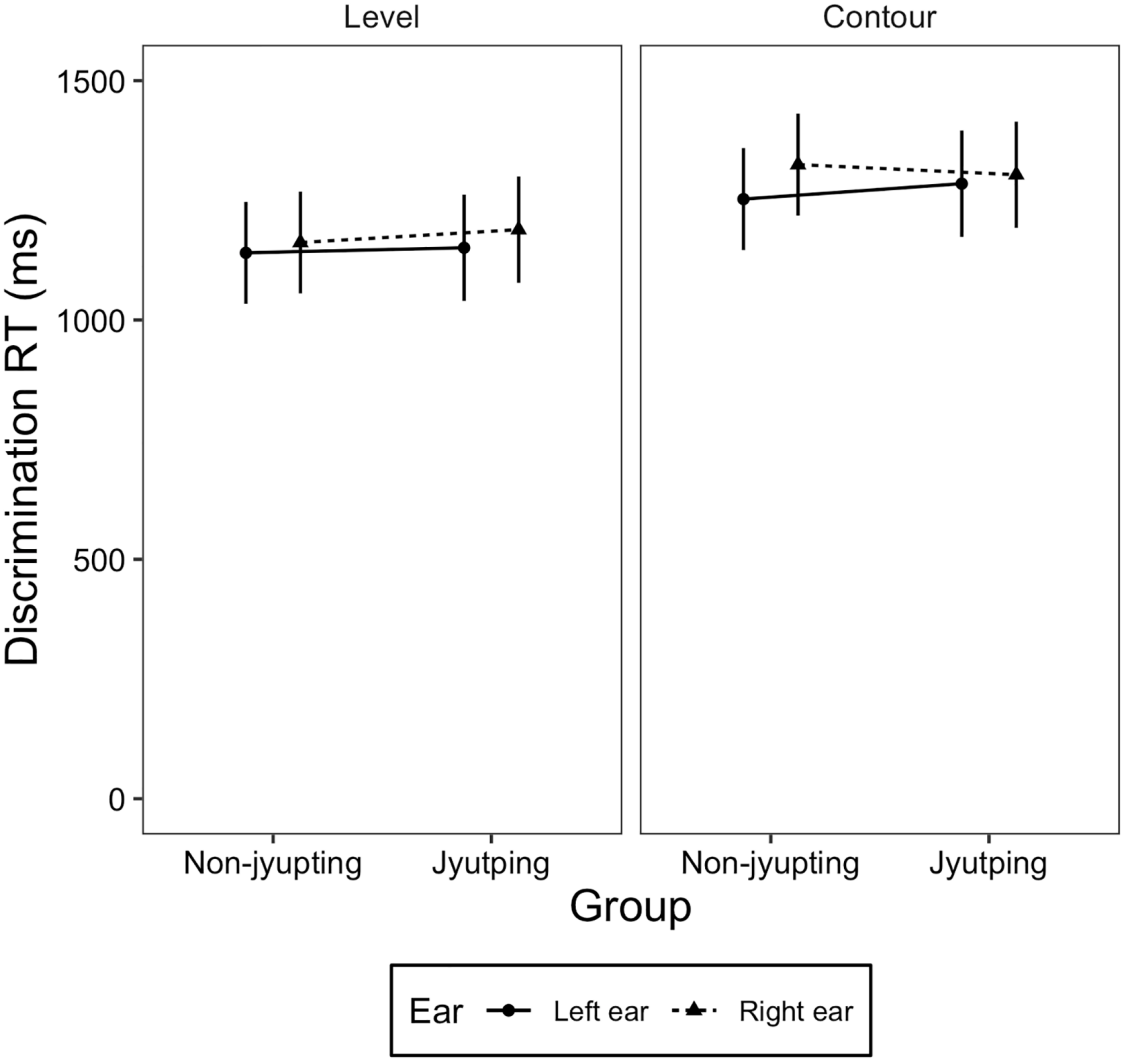
A plot displaying the three-way interaction among *group*, *ear* and *tone type* in the discrimination RT. Discrimination RT were shown for the level and contour tones presented in the left and the right ear in the *Jyutping* and non-*Jyutping* participants. The error bars indicate 95% confidence interval.

We observed that the impact of *Jyutping* expertise on ear preference further interacted with *tone type* on discrimination RT, as shown in a significant three-way interaction among *group*, *ear* and *tone type* [*F* (7, 41.146) = 5.56, *p* = 0.001]. The interaction was plotted in Figure 4. Linear mixed-effect models were fitted within each *tone type* to explore this three-way interaction. For the slowly-changing level tones, there was only a main effect of *ear* [*χ*^2^(1) = 13.101, *p* = 0.002], where the mean RT on the stimuli presented in the left ear was shorter than that on the stimuli presented to the right ear. For the fast-changing contour tones, there was a main effect of *ear* [*χ*^2^(1) = 38.089, *p* < 0.001] and a significant two-way interaction between *ear* and *group* [*χ*^2^(1) = 12.153, *p* < 0.001]. Pairwise comparisons showed that the non-*Jyutping* group exhibited significantly shorter RT in the left ear than the right ear (Estimate = −0.0246, Std. Error = 0.003, *t* = −6.896, *p* < 0.001). This LEA was no longer significant in the *Jyutping* group (Estimate = −0.006, Std. Error = 0.003, *t* = −1.754, *p* > 0.05).

No three-way interaction involving *ear*, *group* and the other two factors was observed in the other analyses (*p*s > 0.05).

### Task difference in the ear preference pattern and general impacts of Jyutping expertise, linguistic-processing demand and tone type

This last section presents the remaining significant effects. Although not a central aim of this study, we observed different ear preference patterns in the identification and discrimination tasks. In addition, we found main and interaction effects that were unrelated to the ear preference issue, but they allowed us to verify whether lexical tone processing performance is influenced by *Jyutping* expertise, linguistic-processing demand (*stimulus type*) and *tone type* as we initially assumed.

Regarding the task difference in the ear preference pattern, the analyses on discrimination accuracy (Figure 1D) revealed a main effect of *ear* [*χ*^2^(1) = 8.762, *p* = 0.003]. Discrimination accuracy on stimuli presented in the left ear was significantly higher than that on stimuli presented in the right ear, suggesting an overall LEA in this task. In contrast, there was no significant main effect of *ear* in the identification accuracy (*p*s > 0.05, Figures 1A,B).

**Figure 5:**
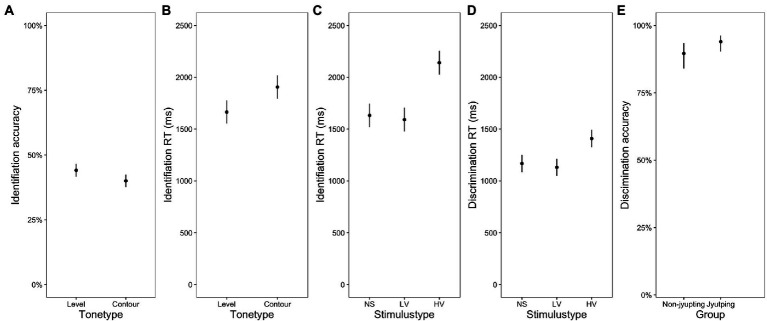
Plots displaying the general effects of *Jyutping* expertise, processing demand and tone type without interaction with *ear*. **(A)** Identification accuracy and **(B)** Identification RT in the contour and level tone conditions; **(C)** Identification RT in the *Jyutping* and non-*Jyutping* participants in the nonspeech tone (NS), low variation (LV) and high variation (HV) conditions; **(D)** Discrimination RT in the nonspeech tone (NS), low variation (LV) and high variation (HV) conditions; **(E)** Discrimination accuracy in *Jyutping* and non-*Jyutping* participants. The error bars indicate 95% confidence interval.

As for the remaining effects, there was a significant main effect of *group* [*χ*^2^(1) = 6.865, *p* = 0.008] in the discrimination accuracy (Figure 5E), with the *Jyutping* group showing higher discrimination accuracy. As for the effects of *tone type*, there was a significant main effect of *tone type* [*F* (1, 406) = 5.497, *p* = 0.01] in the identification accuracy (Figure 5A), with level tones showing higher accuracy scores than contour tones. We also observed a significant main effect of *tone type* [*F* (1, 14.9) = 5.987, *p* = 0.02] in the identification RT (Figure 5B), where level tones elicited shorter RT than contour tones. These effects concerning the tone type are consistent with what was previously reported in the literature (Jia et al., 2013). Finally, with regard to the effects of *stimulus type*, we observed a significant two-way interaction between *stimulus type* and [*F* (1, 35) = 31.46, *p* < 0.001] in the identification RT. Despite this significant interaction, pairwise comparisons with Bonferroni correction found that the group difference was not significant in any stimulus type (*p*s > 0.05). As illustrated in Figure 5C, in both groups, the RT elicited in the high variation condition was significantly longer than that in the low variation and nonspeech tone conditions (*p*s < 0.001), whereas the difference between the low variation and nonspeech tone conditions was not significant (*p* > 0.05). In addition, there was a main effect of *stimulus type* [*F* (2, 32) = 50.88, *p* < 0.001] in the discrimination RT. As shown in Figure 5D, RT elicited in the low variation condition was the shortest, followed by the nonspeech tone condition and then the high variation condition (*p*s < 0.01).

### Summary

Regardless of ear preference, the global performance was in accordance with our expectations: *Jyutping* expertise was associated with higher accuracy in the tone discrimination task; processing speech tones in a high variation context was more challenging than processing speech tones in a low variation context or processing non-speech tones, lengthening the RT in both identification and discrimination tasks; identifying level tones elicited higher accuracy and shorter RT than identifying contour tones.

Regarding the effect of *Jyutping* expertise on ear preference, the non-*Jyutping* group showed an LEA whereas the *Jyutping* group showed an REA in the identification RT, implying different lateralization patterns between the two groups (Figure 1A). Moreover, the discrimination RT results showed an LEA in both groups when they processed the level tones. When they processed the contour tones, an LEA was observed in the non-*Jyutping* group but disappeared in the *Jyutping* group (Figure 4). Nevertheless, both groups showed an LEA in the discrimination accuracy scores (Figure 1D).

The analysis of the effect of linguistic-processing demand on ear preference showed no ear preference in the identification task on the accuracy data. In the discrimination task, the LEA was observed on the accuracy scores, although it was restricted to the nonspeech tone and low variation conditions, while the high variation condition showed a bilateral pattern (Figure 3D).

## Discussion

Ear preference and the underling brain lateralization for lexical tone processing remain an issue of debate. As mentioned in the introduction, in addition to functional and acoustic explanations, the complex patterns of ear preference could be driven by the experience with written codes of lexical tones and the demand on linguistic processing. Additionally, acoustic differences between level and contour tones may also have an impact on brain literalization. To examine these questions, we used a dichotic listening paradigm to investigate the effects of *Jyutping* expertise (*Jyutping* vs. non-*Jyutping* group), linguistic-processing demand (nonspeech vs. low syllable variation vs. high syllable variation), and tone type (level vs. contour tones) on the ear preference pattern in lexical tone processing in Hong Kong Cantonese speakers. In the text below we first discussed the results regarding the effects of these three factors as well as their interactions, followed by a general discussion in the end.

### The effect of Jyutping expertise on ear preference in lexical tone processing

We found that *Jyutping* expertise contributes to some extent to ear preference of lexical tone processing. In the discrimination task, there was a shift from the LEA in the non-*Jyutping* group to a bilateral pattern in *Jyutping* group. However, this shift was observed only in the RT data and during the discrimination of contour tones, which was hypothesized to preferentially rely on the LH auditory cortex based on the temporal integration window hypothesis (Poeppel, 2003; Boemio et al., 2005; Sanders and Poeppel, 2007; Teng et al., 2016; Flinker et al., 2019), but not in level tone discrimination (Figure 4; see the text below for further discussion of the interaction of *Jyutping* expertise and tone type). In the identification task, a more pronounced REA, which suggests a stronger engagement of the LH in lexical tone processing, clearly emerged in the *Jyutping* group. This pattern was observed in the RT but not accuracy, in that the *Jyutping* group showed shorter RTs to stimuli presented in the right ear than the left ear, whereas non-*Jyutping* participants showed shorter RTs in response to stimuli presented in the left ear (Figure 1A). Interestingly, in the identification task, the REA observed in the *Jyutping* group was generalized to all stimulus types and tone types, implying that individuals with *Jyutping* knowledge might have recruited the LH more systematically during lexical tone processing in the identification task.

### The effect of linguistic-processing demand on ear preference in lexical tone processing

A piece of evidence for the influence of linguistic-processing demand on ear advantage is the observation that the LEA found in discrimination accuracy was restricted to the nonspeech tone and low variation conditions, whereas no ear advantage was found in the high variation condition (Figure 3D). In both nonspeech tone and low variation conditions, tone discrimination could be performed based on acoustic processing. Pitch or tone processing in such contexts may mainly recruit the RH, which is thought to predominantly process pitch information (Zatorre and Belin, 2001; Zatorre et al., 2002; Poeppel, 2003). However, in the high variation condition, tones were carried by different syllables. This increase in syllable variability would make the comparison of tones at the purely acoustic/phonetic level difficult. As a result, more effort in linguistic analysis of the speech signal was required, including the separation of tonal information from the segmental elements before performing a tonal comparison. Indeed, high syllable variability led to a significant increase of RTs compared to the nonspeech and low variation conditions (Figures 5C,D). The segmentation process that puts more demand on linguistic processing might entail an increase of activity in the LH spoken language network (Burton et al., 2000). The greater involvement of the LH presumably resulted in bilateral processing of lexical tones in the high variability condition found in the current study, in contrast to the LEA observed in the situations where no segmentation was required. However, we might have to be cautious when interpreting the patterns obtained in the high syllable variation condition. Since segmental variation is present in this condition but not in others, it might have had some contribution to the ear preference pattern in this condition. Future studies that tease apart the influence of segmental variation can shed more light on the effect of linguistic-processing demand on the brain lateralization of lexical tone processing.

### The impact of the interaction between Jyutping expertise and tone type on ear preference in lexical tone processing

Consistent with the extant literature (Jia et al., 2013), we found that level tones were identified more accurately than contour tones (Figures 5A,B). As mentioned in the introduction, lexical tones are characterized by multiple acoustic features and vary along more than one dimension (e.g., pitch height and contour; Gandour, 1983; Chandrasekaran et al., 2007a). Whereas pitch height is the primary cue for distinguishing the three level tones with discernible pitch differences from the beginning of the F0 curve, both pitch height and direction are involved in contour tone distinction and F0 cues in the later portion of the F0 curve might be more critical for contour tone perception (Khouw and Ciocca, 2007). These differences may explain why the participants showed lower accuracy and longer RT when processing Cantonese contour tones compared to level tones.

We also found a complex interaction effect between *Jyutping* expertise and tone type on the ear preference pattern in the discrimination RT (Figure 4). For the level tones, the discrimination RT exhibited an LEA for both groups of participants, whereas for the contour tones, the LEA remained in the non-*Jyutping* group but disappeared in the *Jyutping* group. In other words, only the *Jyutping* group exhibited bilateral processing in the discrimination RT of contour tones. We hypothesized in the introduction that the fast-changing and dynamic contour tones would require the extraction of pitch information over short temporal windows. Moreover, contour tone perception does not only rely on pitch height at the onset, but also on pitch changes in later portions of the pitch curve. These two factors may lead to deeper processing of contour tones and therefore more LH processing, compared to the slowly-changing and static level tones (Poeppel, 2003; Boemio et al., 2005; Sanders and Poeppel, 2007; Teng et al., 2016; Flinker et al., 2019). In line with the discussion here, the group difference we found in the discrimination RT of contour tone processing may suggest that the effect of *Jyutping* expertise was more prominent in the processing of tonal features that preferentially rely on the LH auditory cortex.

### General discussion

As mentioned in the introduction, previous dichotic listening studies on the brain specialization of lexical tones have found conflicting results, reporting three distinct patterns: (1) an REA in processing lexical tones by native Mandarin Chinese, Thai and Norwegian speakers (Van Lancker and Fromkin, 1973, 1978; Moen, 1993; Wang et al., 2001); (2) bilateral processing by native Mandarin Chinese speakers (Baudoin-Chial, 1986); (3) an LEA in the perception of lexical tones by native Hong Kong Cantonese speakers (Jia et al., 2013). The last pattern in Hong Kong Cantonese not only deviates from those observed in other tonal language speakers, but also from an EEG study on Cantonese that revealed left hemispheric lateralization of lexical pitch and acoustic pitch processing, as indexed by the mismatch negativity (MMN; Gu et al., 2013). It also differs from another MMN study which suggests an absence of brain specialization in the processing of Cantonese lexical tones (Jia et al., 2015). These discrepancies warrant more empirical studies.

In order to explain the aforementioned complex results of ear preference in native tone processing, we postulated that a native alphabetic script with codes for lexical tones might play a role. The current study is a first attempt to empirically test this hypothesis and provided some crucial evidence in this direction. Even though the Cantonese speakers in the *Jyutping* group in the current study learned *Jyutping* after childhood, they exhibited either greater REA or a bilateral pattern in the identification task and contour tone discrimination, respectively. The finding suggests that even late acquisition of codes of lexical tones can shape the ear preference, and presumably, underlying brain lateralization, to some extent. Our observation was indeed consistent with other studies reporting that alphabetic literacy enhanced the LH participation in speech processing even in individuals who became literate in adulthood (Dehaene et al., 2010).

It is worth noting that the contribution of *Jyutping* knowledge to ear preference reported here was found on the processing speed but not on response accuracy. This result may be attributable to the fact that RT is a more sensitive measure in capturing the processing advantage of *Jyutping* participants in lexical tone processing ability. Another explanation is related to the relatively late learning of *Jyutping* in Cantonese speakers, unlike in Mandarin Chinese speakers who learn *Pinyin* at the beginning of primary school or even in kindergartens. It is possible that the late acquisition of tonal codes might have a weaker impact on the hemispheric lateralization of native tone processing compared to early acquisition when children’s phonological system is still developing. Lastly, the fact that *Jyutping* uses abstract numbers (1–6) to represent Cantonese lexical tones may also have played some role in the relatively weak impact of *Jyutping*. In contrast, *Pinyin* employs diacritics that contains explicit visual–spatial representations of the pitch information, which has been reported to facilitate Mandarin lexical tone learning (Morett and Chang, 2015). These explanations are not mutually exclusive.

Note that even though dichotic listening paradigm has been long used to investigate brain lateralization in auditory processing, several factors should be taken into consideration in the experimental design to ensure the reliability and validity (Voyer, 1998; Westerhausen, 2019; Westerhausen and Samuelsen, 2020). These factors include stimulus characteristics, stimulus-presentation features, response collection and instruction, participants variables and generalizability (Westerhausen, 2019). In terms of stimulus characteristics, the selection of stimulus materials (e.g., numeric words, non-numeric words, and non-word syllables) should be based on the consideration of processing stages that are of interest (Westerhausen, 2019). In our study, the aim is to investigate the influence of linguistic processing on ear preference. We used nonspeech stimuli and real words which have been widely used to investigate brain specialization and are suitable to investigate our hypothesis. In terms of stimulus-presentation features, the number of 90 to 120 trials seems to be ideal for the reliability and the intensity level of 70 to 80 dB is most commonly used (Westerhausen, 2019). In the current study, the sound level is within the suggested range. In order control the experimental length and avoid fatigue, 54 trials were presented in each block in identification task and 72 trials in each condition in discrimination task. Future studies should increase trial numbers to achieve the reliability. Regarding response collection and instruction, it was found that both verbal and manual responses seem suitable to collect accuracy data (Westerhausen, 2019). The current study required the participants to respond by pressing keys on the keyboard. Regarding the participants, one most important factor is hearing ability, especially the absence of significantly difference in hearing acuity between the two ears should be ensured in the aging population (Westerhausen, 2019). The participants in our study are all young college students and none of them reported hearing problem. However, future studies need to measure the hearing threshold carefully to rule out the possible influence of hearing acuity.

In conclusion, our findings further expanded the understanding of the brain lateralization of lexical tones and addressed some discrepancies in the literature. They suggest that, even among native tonal language speakers, the hemispheric lateralization pattern is not a fixed process but could be influenced by listeners’ phonological skills that are induced or boosted by alphabetic literacy, and by the processing demand inherent to different degrees of linguistic processing as well as acoustic features of lexical tones. The current study also left open several questions to be addressed in future studies. First, it remains to be investigated whether the *Jyutping* participants resorted to the abstract labels of lexical tones while they performed the tasks (e.g., 1–6 in the *Jyutping* transcription), that is the observed impact of Jyutping knowledge would reflect the surface connection between the written and spoken codes of the lexical tone, or whether tone awareness boosted by *Jyutping* skills has a profound influence by progressively restructuring and fine-tuning the phonological representations of tones in Cantonese speakers (Perre et al., 2009; Pattamadilok et al., 2010; Brennan et al., 2013). In the latter case, group differences may be found in tasks that probe phonological representations, such as categorical perception. Although both mechanisms might have contributed to promoting the REA and bilateral pattern observed here, their relative role can be further investigated. Second, it is unknown how much experience with an alphabetic script with tonal codes is necessary to alter the hemispheric laterality of lexical tone processing. In other words, future studies should examine when a shift from the RH dominance to bilateral processing or LH dominance takes place in native speakers when they learn the codes of tones. A training study conducted on Cantonese-speaking adults where their hemispheric laterality will be measured before and after learning tonal codes could provide an approach to address this issue. In relation to this point, a training study like this can also provide more evidence for the causal effect of learning tonal codes on the hemispheric lateralization of native tone processing, because the current study, which is correlational in nature, cannot exclude pre-existing differences between *Jyutping* and non-*Jyutping* participants. In line with this interindividual variation issue, as all the participants (in the *Jyutping* as well as non-*Jyutping* group) are college students in Hong Kong, they are bi-literate in both logographic Chinese and English. It begs the question of whether the participants’ alphabetic English knowledge has any influence on their lexical tone processing and ear preference patterns. However, since the focus of this study is on lexical tone processing and its ear preference pattern, it is highly unlikely that knowledge of the English alphabetic script would affect lexical tone perception. Indeed, previous research suggested that the influence of knowledge of the English alphabetic script on phonological awareness skills in native spoken language processing is limited (Brennan et al., 2013; Zhang Y. et al., 2021). This argument is also partially corroborated by a large amount of cross-linguistic studies that demonstrated the challenge faced by native English speakers when processing or learning lexical tones (Gandour, 1983; Chandrasekaran et al., 2007b; Wong et al., 2007; Qin and Jongman, 2016). A similar issue could be raised regarding the level of proficiency in Chinese. In the present study, we did not measure the participants’ level of Chinese proficiency, which was expected to be relatively high since all participants were native speakers of Chinese. Although unlikely, we cannot objectively rule out the possibility that the *Jyutping* group might somehow have a higher level of Chinese proficiency than the control group, and this potential difference could contribute to their ear preference patterns. Overall, while the hypotheses regarding linguistic-processing demand and tone type are well informed by the literature, a caveat is that these hypothesized operations may not be what actually happened in the listeners’ brain. There may be other sources of individual variance in the processing strategies or mechanisms that future studies should look into. In the present dataset, the analyses were conducted on a relatively small sample size (*N* = 16 in the *Jyutping* group; *N* = 18 in the non-*Jyutping* group). The COVID-19 pandemic has created a difficult environment for recruiting a large sample of participants, especially those well matched on demographic characteristics. Future studies with a larger sample size should try to replicate the current results and obtain more clear-cut ear preference patterns.

## Data availability statement

The datasets presented in this study can be found in online repositories. The names of the repository/repositories and accession number(s) can be found at: https://osf.io/g79sm/.

## Ethics statement

The studies involving human participants were reviewed and approved by The Human Subjects Ethics Sub-committee of The Hong Kong Polytechnic University. The patients/participants provided their written informed consent to participate in this study.

## Author contributions

JS and CZ contributed to conception of the study. JS designed the study and performed the statistical analysis. JS, GZ, and YZ contributed to the data collection. JS wrote the first draft of the manuscript. CZ wrote sections of the manuscript. CZ and CP revised the manuscript. All authors contributed to the article and approved the submitted version.

## Funding

This work was supported by grants from the Departmental General Research Funds (P0008738; https://www.polyu.edu.hk/cbs/web/en/), the Research Grants Council of Hong Kong (ECS: 25603916; https://www.ugc.edu.hk/eng/rgc/), the National Natural Science Foundation of China (NSFC: 11504400; http://www.nsfc.gov.cn/), the Departmental Reward Scheme for Research Publications in Indexed Journals (https://www.polyu.edu.hk/cbs/web/en/) and the Hong Kong Polytechnic Univerity Project of Strategic Importance Scheme (https://www.polyu.edu.hk/en/rio/about-rio/committees/areas-of-excellence-committee/) to CZ. The funders had no role in study design, data collection and analysis, decision to publish, or preparation of the manuscript.

## Conflict of interest

The authors declare that the research was conducted in the absence of any commercial or financial relationships that could be construed as a potential conflict of interest. The reviewer Y-KT declared a shared affiliation, with no collaboration, with the authors to the handling editor at the time of the review.

## Publisher’s note

All claims expressed in this article are solely those of the authors and do not necessarily represent those of their affiliated organizations, or those of the publisher, the editors and the reviewers. Any product that may be evaluated in this article, or claim that may be made by its manufacturer, is not guaranteed or endorsed by the publisher.

## Footnotes

^1^Item was not treated as a random factor in the model because identification accuracy was a set of calculated data which was averaged across items (tone pairs). First, the response to each trial was categorized as either accurate in the left ear or the right ear, or inaccurate. For example, in one trial, the right ear was presented with T1 and the left ear was presented with T3. If a participant’s response was T1, this trial was considered as correct in the right ear, and if the response was T3, it was considered as correct in the left ear; if the response was neither T1 nor T3, it was deemed as incorrect. Identification accuracy was then computed as the relative portion of correct responses in each ear; during this process, item (tone pair) info was not preserved.

^2^As the stimulus set contained tones T3-T6/T6-T3 and T2-T5/T5-T2, which are currently undergoing tone merger in some native speakers of Hong Kong Cantonese, a separate data analysis was conducted excluding these tone pairs. Another separate data analysis was conducted excluding the syllable /fɐn/, the only syllable in the stimulus set (/fɐn/, /jɐu/, and /wɐi/) that does not carry F0 at the syllable onset. Both sets of additional data analyses yield patterns that are qualitatively identical to those reported in the paper.

^3^Additional Bayesian analyses: For the four sets of analyses reported above, the mixed-effects models revealed significant interactions between *group* and *ear* on identification and discrimination RT, but not on identification and discrimination accuracy. To further test the null effects (H0), we conducted Bayesian two-way ANOVA (group by ear) with JASP (JASP Team, 2019). The dependent variable was identification and discrimination accuracy, respectively. For the identification accuracy, Bayesian analysis revealed a BF_01_ value of 25.381, which means that the data were approximately 25 times more likely to occur under the H0 (null hypothesis) than under the H1 (the alternative hypothesis). The error percentage was 1.397%, which reflects the stability of the numerical algorithm that was used to obtain the results. As for the discrimination accuracy, BF_01_ value was 5.005, meaning that the data were approximately 5 times more likely to occur under the H0 than under the H1, with an error percentage of 1.8%. Altogether, these Bayes factors provided additional support for the possibility that the absence of interaction effects in the analysis on identification and discrimination accuracy might not be due to insufficient power.
