# Supplementary material for: The effects of alphabetic literacy, linguistic-processing demand and tone type on the dichotic listening of lexical tones
Source: Front Psychol. 2022 Jul 26;13:877684. doi: 10.3389/fpsyg.2022.877684 (PMC9360803; doi:10.3389/fpsyg.2022.877684)
Supplement: Supplementary file 1 [file Data_Sheet_1.docx]

Supplementary table 1: LME analysis of identification accuracy.

| Fixed Effects | | | | | | | |
| --- | --- | --- | --- | --- | --- | --- | --- |
|  | Estimate | | SE | df | | t | p |
| Intercept | 0.400 | | 0.012 | 406 | | 32.561 | < 0.001*** |
| TonetypeLevel | 0.040 | | 0.017 | 406 | | 2.345 | 0.0195* |
| Random Effects | | | | | | | |
|  | | Variance | | | Std. Dev. | | |
| Participant (Intercept) | | 0.00 | | | 0.00 | | |
| Residual | | 0.03 | | | 0.175 | | |

Supplementary table 2: LME analysis of identification RT.

| Fixed Effects | | | | | | | |
| --- | --- | --- | --- | --- | --- | --- | --- |
|  | Estimate | | SE | df | | t | p |
| Intercept | 3.31 | | 0.002 | 37.34 | | 127.175 | < 0.001*** |
| TonetypeLevel | -0.007 | | 0.003 | 14.93 | | -2.447 | 0.027* |
| StimulustypeNS:GroupNon-jyutping | 0.012 | | 0.003 | 42.06 | | -3.923 | <0.001*** |
| StimulustypeLV:GroupNon-jyutping | -0.129 | | 0.003 | 45.83 | | -4.719 | <0.001*** |
| StimulustypeHV:GroupNon-jyutping | 0.028 | | 0.024 | 34.40 | | 0.929 | 0.359 |
| StimulustypeNS:GroupJyutping | -0.095 | | 0.018 | 31.56 | | -5.29 | <0.001*** |
| StimulustypeLV:GroupJyutping | -0.09 | | 0.015 | 32.02 | | -5.655 | <0.001*** |
| GroupNon-jyutping:EarRight ear | 0.009 | | 0.007 | 5957 | | 1.381 | 0.167 |
| GroupJyutping:EarRight ear | -0.017 | | 0.007 | 5882 | | -2.473 | 0.01* |
| Random Effects | | | | | | | |
|  | | Variance | | | Std. Dev. | | |
| Participant (Intercept) | | 0.124 | | | 0.111 | | |
| TonetypeLevel | | 0.004 | | | 0.069 | | |
| StimulustypeLV | | 0.004 | | | 0.065 | | |
| StimulustypeHV | | 0.004 | | | 0.006 | | |
| Tonepairs (Intercept) | | 0.001 | | | 0.04 | | |
| Residual | | 0.031 | | | 0.75 | | |

Supplementary table 3: LME analysis of discrimination accuracy.

| Fixed Effects | | | | | | | |
| --- | --- | --- | --- | --- | --- | --- | --- |
|  | Estimate | | SE | z | | p |  |
| Intercept | 2.441 | | 0.306 | 7.963 | | < 0.001*** |  |
| GroupJyutping | 0.508 | | 0.198 | 2.576 | | 0.01*** |  |
| EarRightear | -0.326 | | 0.100 | -3.254 | | 0.001*** |  |
| EarLeftear:StimulustypeLV | 1.197 | | 0.199 | 5.990 | | <0.001*** |  |
| EarRightear:StimulustypeLV | 1.097 | | 0.186 | 5.889 | | <0.001*** |  |
| EarLeftear:StimulustypeHV | -0.749 | | 0.171 | -4.382 | | <0.001*** |  |
| EarRightear:StimulustypeHV | -0.448 | | 0.167 | -2.909 | | <0.01** |  |
| Random Effects | | | | | | | |
|  | | Variance | | | Std. Dev. | | |
| Participant (Intercept) | | 0.544 | | | 0.738 | | |
| StimulustypeLV | | 0.594 | | | 0.771 | | |
| StimulustypeHV | | 0.626 | | | 0.791 | | |
| Tonetypeleveltone  Tonepairs (Intercept) | | 0.189  1.122 | | | 0.434  1.105 | | |
| GroupJyutping | | 0.188 | | | 0.433 | | |

Supplementary table 4: LME analysis of discrimination RT.

| Fixed Effects | | | | | | | |
| --- | --- | --- | --- | --- | --- | --- | --- |
|  | Estimate | | SE | df | | t | p |
| Intercept | 3.023 | | 0.02 | 51.99 | | 146.837 | < 0.001*** |
| StimulustypeLV | -0.013 | | 0.006 | 33.108 | | -2.159 | < 0.03* |
| StimulustypeHV | 0.007 | | 0.009 | 33.222 | | 8.388 | <0.001*** |
| GroupNon-jyutping:TonetypeContour:EarLeft ear | 0.041 | | 0.026 | 52.220 | | 1.574 | 0.121 |
| GroupJyutping:TonetypeContour:EarLeft ear | 0.05 | | 0.017 | 23.089 | | 2.903 | 0.007** |
| GroupNon-jyutping:TonetypeLevel:EarLeft ear | -0.012 | | 0.022 | 37.417 | | -0.535 | 0.59 |
| GroupJyutping:TonetypeLevel:EarLeft ear | -.140 | | 0.007 | 41.030 | | -1.793 | <0.08 |
| GroupNon-jyutping:TonetypeContour:EarRight ear | 0.065 | | 0.02 | 48.324 | | 2.421 | 0.01* |
| GroupJyutping:TonetypeContour:EarRight ear | 0.054 | | 0.015 | 16.756 | | 3.416 | 0.03** |
| GroupNon-jyutping:TonetypeLevel:EarRight ear | -0.001 | | 0.02 | 33.695 | | -0.088 | 0.930 |
| Random Effects | | | | | | | |
|  | | Variance | | | Std. Dev. | | |
| Participant (Intercept) | | 0.005 | | | 0.07 | | |
| EarRightear | | 0.0007 | | | 0.027 | | |
| StimulustypeLV | | 0.001 | | | 0.033 | | |
| StimulustypeHV | | 0.002 | | | 0.05 | | |
| Tonepairs (Intercept) | | 0.0007 | | | 0.027 | | |
| GroupJyutping | | 0.00005 | | | 0.007 | | |
| Residual | | 0.012 | | | 0.110 | | |
